# Therapeutic efficacy of artemether–lumefantrine for the treatment of uncomplicated falciparum malaria in northwest Benin

**DOI:** 10.1186/s12936-016-1091-2

**Published:** 2016-01-22

**Authors:** Aurore Ogouyèmi-Hounto, Christian Azandossessi, Souliatou Lawani, Georgia Damien, Yolande Sissinto Savi de Tove, Franck Remoue, Dorothée Kinde Gazard

**Affiliations:** Unité d’Enseignement et de Recherche en Parasitologie Mycologie/Faculté des Sciences de la Santé, laboratoire du Centre de Lutte intégrée contre le paludisme, 01 BP188 Cotonou, Benin; Laboratoire du Centre de Lutte intégrée contre le paludisme, 01 BP188 Cotonou, Benin; UMR 224-MIVEGEC, Institut de Recherche pour le Développement, 08 BP 841 Cotonou, Benin

**Keywords:** Efficacy, Artemether–lumefantrine, Falciparum malaria, Benin

## Abstract

**Background:**

Artemether/lumefantrine (Coartem^®^) has been used as a treatment for uncomplicated *Plasmodium falciparum* infection since 2004 in Benin. This open-label, non-randomized study evaluated efficacy of artemether–lumefantrine (AL) in treatment of uncomplicated falciparum malaria in children aged 6–59 months in two malaria transmission sites in northwest Benin.

**Methods:**

A 42-day therapeutic efficacy study was conducted between August and November 2014, in accordance with 2009 WHO guidelines. One-hundred and twenty-three children, aged 6 months to 5 years, with uncomplicated falciparum malaria were recruited into the study. The primary endpoint was parasitological cure on day 28 and day 42 while the secondary endpoints included: parasite and fever clearance, improvement in haemoglobin levels. Outcomes were classified as early treatment failure (ETF), late clinical failure, late parasitological failure, and adequate clinical and parasitological response (ACPR).

**Results:**

Before PCR correction, ACPR rates were 87 % (95 % CI 76.0–94.7) and 75.6 %, respectively (95 % CI 67.0–82.9) on day 28 and day 42. In each study site, ACPR rates were 78.3 % in Djougou and 73 % in Cobly on day 42. There was no ETF and after PCR correction ACPR was 100 % in study population. All treatment failures were shown to be due to new infections. Fever was significantly cleared in 24 h and approximately 90 % of parasites where cleared on day 1 and almost all parasites were cleared on day 2. Haemoglobin concentration showed a slight increase with parasitic clearance.

**Conclusion:**

AL remains an efficacious drug for the treatment of uncomplicated falciparum malaria in Benin, although higher rates of re-infection remain a concern. Surveillance needs to be continued to detect future changes in parasite sensitivity to artemisinin-based combination therapy.

## Background

Malaria remains a major public health problem in tropical regions. According to the World Health Organization (WHO), in 2014 there were an estimated 198 million cases and 584,000 deaths in children under 5 years of age [1]. In Benin, malaria represents the major reason for clinical consultation and hospitalization [2]. Early diagnosis and prompt treatment of cases are the most important strategies for the control and prevention of this disease. Unfortunately, *Plasmodium falciparum* has developed resistance to numerous anti-malarial drugs such as chloroquine (CQ) and sulfadoxine–pyrimethamine (SP) [3]. Drug resistance can affect efforts to control malaria, and lead to increased malaria-related mortality and morbidity. The WHO now recommends the use of artemisinin-based combination therapy (ACT) for treatment of uncomplicated falciparum malaria in countries where the rates of (CQ/SP) drug resistance are high [4]. ACT is thus widely promoted as a strategy to counteract the increasing resistance of *P. falciparum* to anti-malarial drugs, and to prevent disease transmission through their action against gametocytes [5–7]. In Benin, as a result of increasing failure rates of treatment with CQ and SP, the national anti-malarial drug policy was changed in 2004, with the official withdrawal of CQ and SP replaced by an artemisinin-based combination (artemether–lumefantrine, AL). Currently, ACT remains highly effective with rapid effects on fever in almost all settings as long as the partner drug in the combination is effective. In Africa, several studies demonstrated its efficacy and tolerability [8–10] unlike in some regions of Southeast Asia where delayed response to ACT has been reported [11, 12]. Because of the threat of emergence and spread of artemisinin resistance in malaria-endemic countries, especially in Africa, in vivo anti-malarial efficacy studies are recommended by WHO [13] for monitoring ACT efficacy in all countries where ACT has been deployed, to ensure its long-term usefulness. In Benin, the Ministry of Health, through the National Malaria Control Programme (NMCP), has been routinely conducting therapeutic efficacy tests (TETs) at several sentinel sites located in different parts of the country. The findings of these TETs have provided useful data to support changes of anti-malarial drug policy in Benin, including the change from CQ and SP to AL as stated above. However, the original sentinel sites do not cover all the country’s settings whereas previous studies with other anti-malarial drugs showed that the rate of adequate clinical and parasitological response (ACPR) varies according to location. Furthermore, the programme of routine surveillance with TETs for ACT has not yet been implemented, and in any case does not always include genotypic confirmatory tests. To fill this gap, studies were initiated in two settings in the northwest of Benin, where routine surveillance has never been conducted, in order to provide efficacy data on AL according to a standard WHO 42-day follow-up therapeutic efficacy protocol [14].

## Methods

### Study site, design and target population

The study was conducted in Benin between August and November 2014 in two health centres: Djougou, 450 km from Cotonou (the capital city), and Cobly, 643 km from Cotonou. The distance between the two sites is about 180 km. In the two sites, malaria transmission occurs from May to November during the rainy season. *Plasmodium falciparum* is the predominant parasite species transmitted by *Anopheles gambiae* s.s. (85 %) and *Anopheles arabiensis* (15 %) [15]. Prevalence of falciparum infection was 19.1 % in Djougou and 18 % in Cobly (unpublished data). This study was designed as a prospective, open-label, non-randomized single-arm trial based on the WHO protocol of 2009 [14] and included children aged 6–59 months with uncomplicated falciparum malaria infection.

### Sample size

The sample size was determined based on WHO standard protocol [14]. Considering the unknown proportion of treatment failure rate of AL in study sites, the clinical failure rate was assumed to be 50 %, with confidence level of 95 % and a precision around the estimate of 5 %. A minimum sample of 50 patients was required for the study. With a 20 % increase to allow loss to follow-up and withdrawals during the 42-day follow-up period, 60 patients by site were planned to be included into the study. The study recruited a total of 123 patients overall.

### Screening and recruitment

Children aged six to 59 months who attended the outpatient department of Djougou and Cobly Health Centres were screened, and those who met the eligibility criteria were enrolled into the study. Inclusion criteria included: (1) fever during the past 24 h or fever at presentation (axillary temperature ≥ 37.5 °C); (2) *P. falciparum* mono-infection with parasite density between 2000 and 200,000 parasite asexual forms per μL of blood identified by microscopy on blood smears; (3) no evidence of a concomitant febrile illness; (4) no signs/symptoms of severe malaria as defined by WHO [16]; (5) ability and willingness to attend scheduled follow-up visits and stable residence within the catchment area throughout the study period; and, (6) written informed consent from parents. Patients with presence of severe malnutrition and regular medication which might have interfered with anti-malarial pharmacokinetics were not included but received appropriate treatment according to the national guidelines.

### Laboratory methods

Laboratory screening involved a finger prick to collect blood samples for detection of malaria parasites by rapid diagnostic test (RDT) and microscopy using thick and thin smears. Patients who fulfilled the inclusion criteria and who had positive RDT results were enrolled in the study and sent to the laboratory for collection of blood samples, including two blood slides to detect presence of malaria parasites, the level of parasitaemia, species, by microscopy, dried blood spots (DBS) on filter papers, blood for haemoglobin determination. The filter papers were air-dried and stored in self-sealing plastic bags with desiccators for further molecular analysis.

### Thick and thin smears

Thick and thin blood smears were prepared, stained with 10 % Giemsa for 10–15 min. Parasite density was determined by counting the number of asexual parasites per 200 white blood cells, and calculated per μL using the following formula: numbered parasites × 8000/200 assuming a white blood cell count of 8000 cells/μL [17, 18]. Absence of malaria parasite in 200 high power ocular fields of the thick film was considered as negative. Detection of the different parasite species was done on thin films. All slides were read in the health centres’ laboratory with external quality control of 10 % of the negatives slides and all positives in the Reference Laboratory of Parasitology of the Centre National Hospitalier Universitaire of Cotonou.

### Haemoglobin measurement

Finger-pick blood sample was used to measure haemoglobin using a portable spectrophotometer (Hemo-Control, EKF-Diagnostic Gmbh, Germany).

### Genotyping

Polymerase chain reaction (PCR) analysis was done to distinguish recrudescence from new infection on sample with treatment failure. Nested PCR was conducted on paired samples (parasite collected on day 0 and failure day) to compare two polymorphic genetic markers from *P. falciparummsp1* and *msp2* genes [19, 20]. After DNA extraction, PCR was performed using family-specific primer pairs as previously described [21, 22]. The molecular analyses were performed in the Molecular Biology Laboratory of the Centre de lutte integrée contre le Paludisme.

### Treatment and follow-up

Patients enrolled in the study were treated with AL (Coartem^®^, Novartis Pharmaceuticals, New York, USA for Novartis Pharma AG, Basle, Switzerland, manufactured January 2014, expiry date December 2015, Lot: F1214), a fixed combination of 20 mg of artemether and 120 mg lumefantrine in a tablet. The drugs were administered according to the manufacturer’s recommended dose based on the weight of patient. A full course of AL consisted of six doses given twice daily. Patients were observed for 30 min to ensure that they did not vomit the study drug. When vomiting occurred, a repeat dose was given after vomiting stopped. Any patient who persistently vomited the study drug, i.e., three times, treatment was discontinued and such patient was withdrawn from the study, and treated with parenteral quinine according to the national guidelines for management of complicated malaria. The day a patient was enrolled and received the first dose of AL was designated day 0. Paracetamol was given to all patients with body temperature greater than or equal to 38 °C. All patients were hospitalized within three days (days 0, 1 and 2) and treatment was administered orally under direct observation of a nurse with fatty food or milk. After three days of treatment, patients returned home and follow-up was done for 42 days with scheduled visits on days 3, 7, 14, 21, 28, 35, and 42 or at another day (unscheduled visits) when patient felt unwell. Parents/guardians were informed and encouraged to bring the children to the clinic whenever they were unwell without waiting for their scheduled visits. The study team made home visits as follow-up for study participants that were late for their scheduled visits. Patients who travelled to other places and could not be traced for scheduled follow-up were withdrawn from the study. Patients withdrawn for the re-appearance of *P. falciparum* were treated with quinine 30 mg/kg/day in three doses. During the visits, both clinical and parasitological assessments were performed. The thick smear was performed every 6 h from day 0 to day 2 and once a day during the follow-up (day 3, 7, 14, 21, 28, 35, and 42) and on any unexpected visit by the patient. Haemoglobin measurement was performed at enrolment and day 2, 7, 28, and 42 to assess the influence of anti-malarial treatment on anaemia.

### Outcome classification

The primary endpoint was parasitological cure on day 28 and day 42 as per WHO protocol of 2009 [14] and secondary endpoints included parasite and fever clearance, improvement in haemoglobin levels at day 42 from the day 0 baseline. Treatment outcomes were classified as early treatment failure (ETF), late clinical failure (LCF), late parasitological failure (LPF), and adequate clinical and parasitological response (ACPR) before and after PCR correction [14].

### Data analysis

Data from both clinical and parasitological assessments from the case report for each study participant were entered on an individual record and then into the WHO standardized Microsoft Excel data collection form [14]. This form was used both for data management and analysis. Additional analysis was conducted with Microsoft Excel. The data were entered in the Excel database and verified by another person using the case report form. Treatment outcome was analysed based on Kaplan–Meier analysis.

### Ethical considerations

Ethical clearance for this study was obtained from the National Ethics Committee for Health Research of Ministry of Health. Oral and written informed consent was obtained from parents or guardians of all patients before they were screened for possible inclusion into the study.

## Results

**Fig. 1:**
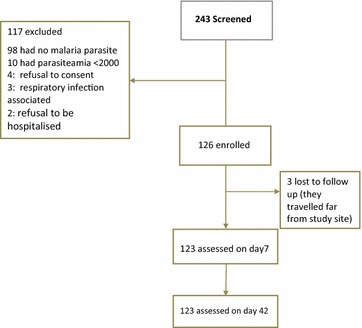
Profile of patients screened and enrolled in the study

A total of 132 and 111 potentially eligible patients from Djougou and Cobly, respectively, were screened for participation in the study. Following application of inclusion criteria, a total of 126 (63 in both sites) were enrolled into the study. The causes of non-inclusion of eligible patients were: no or low parasite density, refusal to consent, other diseases such as respiratory infections, refusal to be hospitalized. Due to three lost-to-follow-up children who travelled out of the study area, between days 3 and 7, the total analysable population was 123 (Fig. 1), comprising 37 % female (45/123) and 63 % (78/123) male participants. Patients’ mean age was 31.3 ± 13.5 months. The mean body temperature at enrolment was 38.7 ± 0.9 °C while the mean haemoglobin concentration was 8.9 ± 2.0 g/dL. The parasite density ranged from 2028 to 192,715 with a mean of 42,329 asexual parasites/μL. Characteristics of the study population are detailed in Table 1.

**Table 1:** Baseline characteristics of study participants

Variable	Overall
Number of patients enrolled (n)	123
Age (months)	6–59 months
Mean (DS)	31.3 ± 13.5
Gender	
Male [n (%)]	78 (63 %)
Female [n (%)]	45 (37 %)
Body temperature [°C, mean (SD)]	38.7 ± 0.9
Range	37.8–41
Parasite density [parasite/μL, mean (SD)]	42,329 ± 50,432
Range	2028–192,715
Haemoglobin [g/dL, mean (SD)]	8.9 ± 2.0
Range	7.2–16.2

### Primary study outcomes

**Fig. 2:**
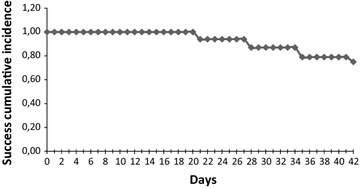
Kaplan–Meier curves showing treatment success cumulative proportion up to day 42 of follow-up PCR-uncorrected

Classification of treatment outcomes (PCR uncorrected and PCR corrected) is presented in Table 2. At day 28, an ACPR was noted in 87 % without PCR correction and in 100 % after PCR correction. On day 42, analysis of PCR uncorrected data estimated ACPR as 75. 6 % (CI 66.5–82.6). No patients showed ETF, while LCF was reported in 24 study participants (19.5 % CI 12.9–27.6), with 17 in Cobly and seven in Djougou. LPF was observed in six (4.9 % CI 1.8–10.3) of the evaluated study population, three cases in Cobly and three in Djougou. Kaplan–Meier survival analysis of the PCR uncorrected data showed estimates of success of 1.00 between days 0 and 20; 0.94 from days 21 to 27; 0.87 from days 28 to 34; 0.79 from days 35 to 41; 0.75 on day 42 (Fig. 2). The PCR corrected cure rate showed that 100 % of the patients had ACPR to AL treatment. All treatment failures were shown to be due to new infections and, therefore the Kaplan–Meier survival analysis of the PCR corrected data showed estimates of success of 1.00 from days 0 to 42, translating into an estimate of cumulative failure incidence of 0.00 from days 0 to 42.

**Table 2:** Treatment outcome

Outcome	Day 28		Day 42	
	PCR uncorrected (IC 95 %)	PCR corrected (IC 95 %)	PCR uncorrected (IC 95 %)	PCR corrected (IC 95 %)
Early treatment failure (ETF)	0	0	0 (0 %) [0.0–3.0]	0 (0 %) [0.0–3.9]
Late clinical failure (LCF)	11 (8.9 %) [4.1–13.7]	0	24 (19.5 %) [12.9–27.2]	0 (0 %) [0.0–3.9]
Late parasitological failure (LPF)	05 (4.1 %) [1.2–9.6]	0	6 (4.9 %) [1.8–10.3]	0 (0 %) [0.0–3.9]
Adequate clinical and parasitological response (ACPR)	107 (87 %) [76.0–94.7]	107 (100 %) [97.2–100]	93 (75.6 %) [67.0–82.9]	93 (100 %) [96.1–100]
Total analysis	123		123	
Withdrawn	0		0	
Loss to follow-up	3 (2.4 %)		3 (2.4 %)	
Total	126		126

### Secondary study outcomes

**Fig. 3:**
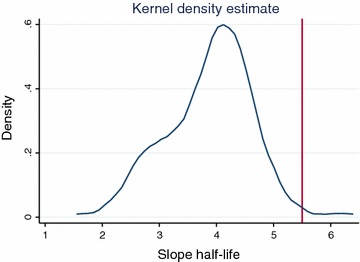
Distribution of parasite clearance half-lives

**Fig. 4:**
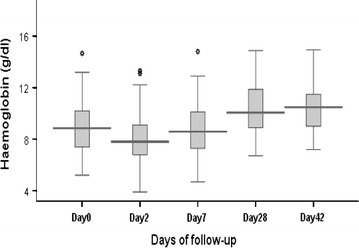
Distribution of haemoglobin recovery

On day 1 post-treatment, 21/123 (17.1 %) slides were negative while 101/123 (82.1 %) were negative on day 2. On day 3 all slides were negative. This rapid decrease of parasitaemia was confirmed by a rapid decline of mean parasitaemia during the first 18 h (H0 = 41,317 P/µl; H6 = 30,431 P/µl; H12 = 15,280 P/µl, H18 = 2631 P/µl); the distribution of parasite clearance half-lives is presented in Fig. 3 (thick films every 6 h). The Figure shows that most individuals had clearance half-lives of around 4 h with two individuals who had a delay in the speed of parasite clearance around 5.5 h. There was a general reduction in fever within 24 h of initiation of treatment, and this was maintained until the end of the 42-day follow-up, indicating a fever clearance time of 24 h. The haemoglobin concentration increased mildly in conjunction with parasitic clearance from the blood, however, the mean change in haemoglobin concentration was statistically significant from days 2 to 7 (P = 0.0113) and days 7 to 28 (P = 0.0000) (Fig. 4).

## Discussion

AL combination was officially introduced for the treatment of uncomplicated malaria in Benin in 2004. At that particular time, the drug was not generally accessible to the population, but it is nowadays distributed to all public health facilities largely through collaborative efforts involving Benin’s NMCP and its technical and financial partners. The study described here was conducted, according to WHO protocol, in children aged 6–59 months, the group most vulnerable to malaria in countries such as Benin where malaria transmission is characterized as stable. Before the age of 5 years children have not developed effective anti-malarial immunity. Importantly, the follow-up period of 42 days, rather than the 28 days frequently used in studies of this type, allows assessment of the impact of new infections arising in each child. In this study an ACPR, without PCR correction, was observed in 75.6 % of the overall population with no significant difference between the two geographically separated study sites (78.3 % Djougou, 73 % Cobly; P = 0.56). This ACPR rate is higher than the 33.3 % obtained by Tinto et al. [23] after 42 days of follow-up in children under 5 years treated with AL, likely the result of a very high rate of re-infection. No cases of ETF were observed in the study presented here. Moreover, the cases of LCF and LPF observed were all found to be the result of re-infections, giving an ACPR of 100 % after PCR correction. This result thus confirms that the efficacy of the AL combination for first-line treatment of uncomplicated falciparum malaria in Beninese children under 5 years of age remains acceptably high after more than 10 years’ use. However, it is important to consider whether using only the markers *msp1* and *msp2* might have contributed to this very low recrudescence rate. It is true that the markers recommended by WHO are *msp1*, *msp2* and *glurp* [24], but these markers should be genotyped sequentially, from the higher to the lowest discriminatory power. Once the analysis of one marker has shown a new infection, the analysis should be stopped. If no evidence of new infection is detected with the first markers, the second marker should be analysed. If no new infection is detected, then the third marker should be used. This would mean that the result could be given with the genotyping of a single marker and the low rate of recrudescence in this study cannot be attributed to the use of only two markers. Furthermore, several studies [25, 26] in which three markers were used also found very low recrudescence rates. Findings in this study are consistent with other therapeutic efficacy studies with AL conducted both in the past in Benin [27, 28] and elsewhere in sub-Saharan African countries (SSA) in which the PCR corrected ACPR ranged from 96 to 100 % [25, 26, 29, 30]. Participants with re-infections in the present study received quinine as a second-line treatment because routine health services in Benin lack the means to distinguish between recrudescences and new infections. However, it remains unclear whether quinine or the same ACT (AL) would be the optimal treatment in such cases. Given that nearly all recurrent parasitaemias were caused by new infections, it is reasonable to imagine that re-treating the child with the same ACT regimen, rather than with quinine, would be appropriate. However, other studies using treatment with AL after 28 or 42 days follow-up noted further recrudescences suggesting drug failure with, respectively, 82.4, 92 and 93 % of ACPR after PCR correction [31–33]. Such cases of recrudescence necessitate evaluation for markers of resistance to detect as early as possible evidence of the occurrence of artemisinin resistance. In the study presented here, a total of 30 cases of LTF were observed from day 21 onwards, giving a rate of re-infection of 24.4 % in the study population. A similarly high re-infection rate following AL treatment was observed in Zambia with 37 % in Chongwe [29], 20.8 % in Chipata [29], 30 % in Ndola [33], and more than 25 % in Burkina Faso [34]. In Mali [35] a study on the efficacy and safety of different ACT found more cases of re-infection with AL than with other ACT, such as artesunate–amodiaquine (AS + AQ), artesunate and sulfadoxine–pyrimethamine (AS + SP). These results raise the question of the efficacy of lumefantrine, the long-acting partner drug in the AL combination that should prevent early re-infections. The half-life of artemisinin is approximately 2 h versus four to six days for lumefantrine [36, 37], which thereby prolongs the antiplasmodial action of the drug combination. Plausibly then, the occurrence of frequent re-infections might indicate a decrease in the sensitivity of some plasmodial strains to lumefantrine. Such results indicate a requirement for regular *in vitro* monitoring of the efficacy of lumefantrine on plasmodial strains in countries where the AL combination is used as first-line treatment. The absence of ETF during treatment with AL in this study and in several others [25, 27, 38, 39] highlights the drug’s efficacy and is emphasized by the rapid rate (48 h) of parasite clearance. These findings are similar to those previously reported in studies from several other countries [27, 33, 40, 41]. AL clears parasites quickly as a result of the rapidly absorbed, fast-acting artemisinin component. Here, parasite clearance half-lives were around 4 h for most individuals with rapid decline of mean parasitaemia during the first 18 h, results similar to those previously reported in other parts of Africa [40–42]. The difference with the study in Nigeria [43], where parasitaemia disappeared in all children after 16 h, could be explained by the fact that the study in Nigeria included children aged 12–132 months, i.e., children aged one to 11 years old, many of whom will have developed immunity that can synergize with drugs to promote the rapid elimination of parasites. In this study, having two individuals with delay in clearance half-lives does not undermine the efficacy of AL. According to WHO, partial resistance to artemisinin is suspected when more than 10 % of patients have a parasite clearance half-life longer than 5 h after treatment with ACT [44].

Rapid fever clearance was noted in all participants, 100 % of whom were fever-free within 24 h. Fever clearance kinetics could also be explained by the fast-acting parasite clearance properties of artemisinins, leading to rapid resolution of symptoms including fever [45]. An antipyretic (paracetamol) was given to febrile patients, however no patients in the study required paracetamol after 24 h. It is nevertheless important to note that the use of paracetamol should be discussed as a confounding factor contributing to fever clearance time of AL. Other studies have reported similar findings [25, 26, 33, 46]. Although AL cleared fever and parasitaemia in a very short period of time (less than three days), a concomitant significant increase in the concentration of haemoglobin was not observed. This less-pronounced post-treatment haematological recovery suggests that malaria could be the major contributing factor to the low haemoglobin levels at enrolment, but that the slow rate of recovery may imply that in SSA countries, other factors, such as geohelminths and malnutrition, may play a key role in the occurrence of anaemia as reported in other studies [40].

## Conclusion

AL remains an effective drug for the treatment of uncomplicated falciparum malaria in Benin although higher rates of re-infection remain a concern. Ten years after its introduction as a first-line drug, AL remains effective, rapidly clearing fever and parasites within 48 h. Regular surveillance should to be continued in these and other sites in the country to provide early warnings of changes in parasite sensitivity to ACT. To reduce the burden of malaria, the efficacy of AL, of other ACT and of their partner drugs needs to be carefully and periodically monitored in order to provide the evidence-base for timely reviews of malaria treatment policy.
